# The Effects of Serum ANGPTL8/betatrophin on the Risk of Developing the Metabolic Syndrome – A Prospective Study

**DOI:** 10.1038/srep28431

**Published:** 2016-06-27

**Authors:** Haoyu Wang, Yaxin Lai, Cheng Han, Aihua Liu, Chenling Fan, Hong Wang, Hongmei Zhang, Shuangning Ding, Weiping Teng, Zhongyan Shan

**Affiliations:** 1 Department of Endocrinology and Metabolism, Institute of Endocrinology, Liaoning Provincial Key Laboratory of Endocrine Diseases, The First Affiliated Hospital of China Medical University, China Medical University, No. 155 Nanjing North Street, Shenyang, Liaoning 110001, China

## Abstract

ANGPTL8/betatrophin is a recently discovered hormone, which mainly synthesized and secreted by liver and adipose tissue, playing a critical role in pancreatic beta cell proliferation. Previous studies have suggested that serum ANGPTL8/betatrophin levels are associated with obesity and diabetes mellitus. Here, we evaluated the prospective association between ANGPTL8/betatrophin and the metabolic syndrome from a community-based cohort of 153 adults without metabolic syndrome. After 3.5-year follow-up, we observed an inverse correlation between the baseline ANGPTL8/betatrophin levels and the incidence of metabolic syndrome, even after multivariate adjustments. In receiver operating characteristic analysis, the area underneath the curve for ANGPTL8/betatrophin was 0.70 in males and 0.86 in females, and the optimal cut-off values were 23.9 ng/mL and 31.1 ng/mL, respectively. This article suggests that ANGPTL8/betatrophin might be useful in predicting newly-onset metabolic syndrome and its progression in clinical setting.

Metabolic syndrome (MetS) is a group of metabolic disorders that leads to a higher risk of type 2 diabetes mellitus (T2DM) and cardiovascular disease^1^,^2^. The major abnormalities of MetS include abdominal obesity, hypertriglyceridemia, low high-density lipoprotein cholesterol (HDL-c) levels, hyperglycemia, hypertension and insulin resistance^3^. Given the rapidly increasing prevalence of the MetS^4^,^5^ and its possible harmful outcome, further research to reveal its predictive factors and mechanisms is necessary.

ANGPTL8/betatrophin, a newly member of angiopoietin-like protein family, which is produced by the hepatic tissue and white adipose tissue, has been reported in several insulin resistance animal models^6^. For the MetS is closely associated with glucose and lipid homeostasis, and the alteration of ANGPTL8/betatrophin has been reported to be involved in proliferation of pancreatic beta cells and regulation of glucose and lipid metabolism in mice^6^,^7^,^8^, ANGPTL8/betatrophin could be a strong candidate for prediction of future development of the MetS.

Recent studies mentioned above evaluated serum concentrations of ANGPTL8/betatrophin in patients with different glucose tolerance levels. However, as far as we know, it is no prospective evidence for the ANGPTL8/betatrophin-MetS association in adults that has been published. Therefore, the present prospective study was conducted to explore the relations of circulating ANGPTL8/betatrophin concentrations with the risk of incident MetS in Chinese adults.

## Results

### General characteristics of study subjects stratified for MetS and non-MetS

After 3.5-year follow-up, 18 males (27.3%) and 10 females (11.5%) developed the MetS. In comparison with the non-MetS group, the baseline body mass index (BMI), waist circumference (WC) and visceral fat (VF) were significantly higher in both males and females who developed the MetS, while no significant differences were observed in age, systolic blood pressure (SBP), diastolic blood pressure (DBP), total cholesterol (TC) as well as HDL-c levels (Table 1). Besides, waist-hip rate (WHR), two-hour postprandial plasma glucose (2hPG), triglyceride (TG), low-density lipoprotein cholesterol (LDL-c), homeostasis model assessment-insulin resistance (HOMA-IR) and subcutaneous fat (SF) were elevated significantly in males who developed the MetS, compared to their healthy controls (all *p *< 0.05).

### Correlation between serum ANGPTL8/betatrophin and metabolic risk parameters

At baseline, our data revealed a positive correlation of ANGPTL8/betatrophin with age (r = 0.31, *p* = 0.012 in males; r = 0.22, *p* = 0.045 in females) in all participants. On the other hand, inverse associations were observed between ANGPTL8/betatrophin and BMI (r = −0.26, *p* = 0.039 in males; r = −0.42, *p* < 0.001 in females), WC (r = −0.32, *p* = 0.010; r = −0.43, *p *< 0.001), WHR (r = −0.35, *p* = 0.004; r = −0.38, *p* < 0.001), SF (r = −0.26, *p* = 0.038; r = −0.33, *p* = 0.002), as well as VF (r = −0.33, *p* = 0.007; r = −0.34, *p* = 0.001). However, the present study showed no correlation between ANGPTL8/betatrophin and SBP, DBP, fasting plasma glucose (FPG), fasting insulin (FINS), 2hPG, HbA1c, HOMA-IR, TG, TC, LDL-c, ALT, AST, and GGT. In addition, correlation analyses demonstrated a negative association of ANGPTL8/betatrophin with HDL-c (r = −0.36, *p* = 0.001) in females, as well as SF/VF in males (r = 0.34, *p* = 0.005).

### Association between serum ANGPTL8/betatrophin levels and the incidence of the MetS

**Figure 1:**
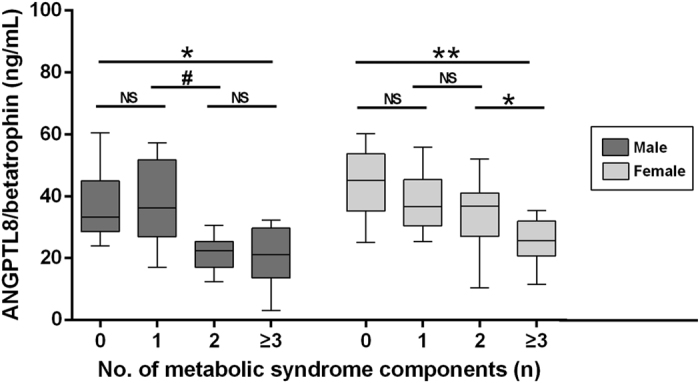
Baseline serum ANGPTL8/betatrophin levels according to the number of MetS components during follow-up. NS, not significant #*p* = 0.013 *p < 0.01 **p < 0.001.

In comparison of non-MetS group, the median ANGPTL8/betatrophin concentrations in the patients suffering from MetS were decreased, in both males (22.93 vs. 29.28 ng/mL, *p* = 0.011) and females (25.46 vs. 38.10 ng/mL, *p *< 0.001). A similar association was observed between ANGPTL8/betatrophin and high blood glucose (Table 2). Furthermore, the serum levels of ANGPTL8/betatrophin were gradually reduced with an increasing number of MetS components during the follow-up period (*p* for trend = 0.005 for males; *p *< 0.001 for females) (Fig. 1).

Multivariate adjusted analyses were further performed separately by gender to uncover the association between ANGPTL8/betatrophin and the incidence of MetS. In males, compared to the lowest quartile of ANGPTL8/betatrophin levels (≤21.28 ng/mL), the odds ratio (OR) of MetS was significantly decreased in subjects in the three higher quartiles (OR, 0.92; 95% CI, 0.85–0.99, *p* = 0.031). The corresponding ORs (95% CIs) for the incident of high WC, low HDL-c, high TG, high blood pressure and high blood glucose were 0.93 (0.82–1.07), 0.91 (0.72–1.15), 0.86 (0.71–1.04), 1.00 (0.94–1.06), 0.74 (0.57–0.96), respectively.

In females, compared to the lowest quartile of ANGPTL8/betatrophin levels (≤29.25 ng/mL), the risk of developing MetS in the three higher quartiles was also decreased (OR, 0.84; 95% CI, 0.74–0.96, *p* = 0.010). The corresponding ORs (95% CIs) for the incident of high WC, low HDL-c, high TG, high blood pressure and high blood glucose were 0.98 (0.76–1.24), 0.96 (0.78–1.18), 0.91 (0.84–0.98), 0.99 (0.94–1.04), 0.85 (0.76–1.01), respectively.

In both males and females, no significant relationship was found in predicting the progression of MetS components, except for high blood glucose and female hypertriglyceridemia (Table 3).

### Receiver operating characteristic analysis of MetS risk

The areas under curve (AUC) of the receiver operating characteristic (ROC) used to assess MetS risk based on ANGPTL8/betatrophin and other metabolic parameters (Table 4). In both males and females, ANGPTL8/betatrophin and WC showed statistically significances in prediction of MetS(all *p *< 0.05). In addition, TG and 2hPG played predictive roles significantly only in males (all *p* < 0.05).

**Figure 2:**
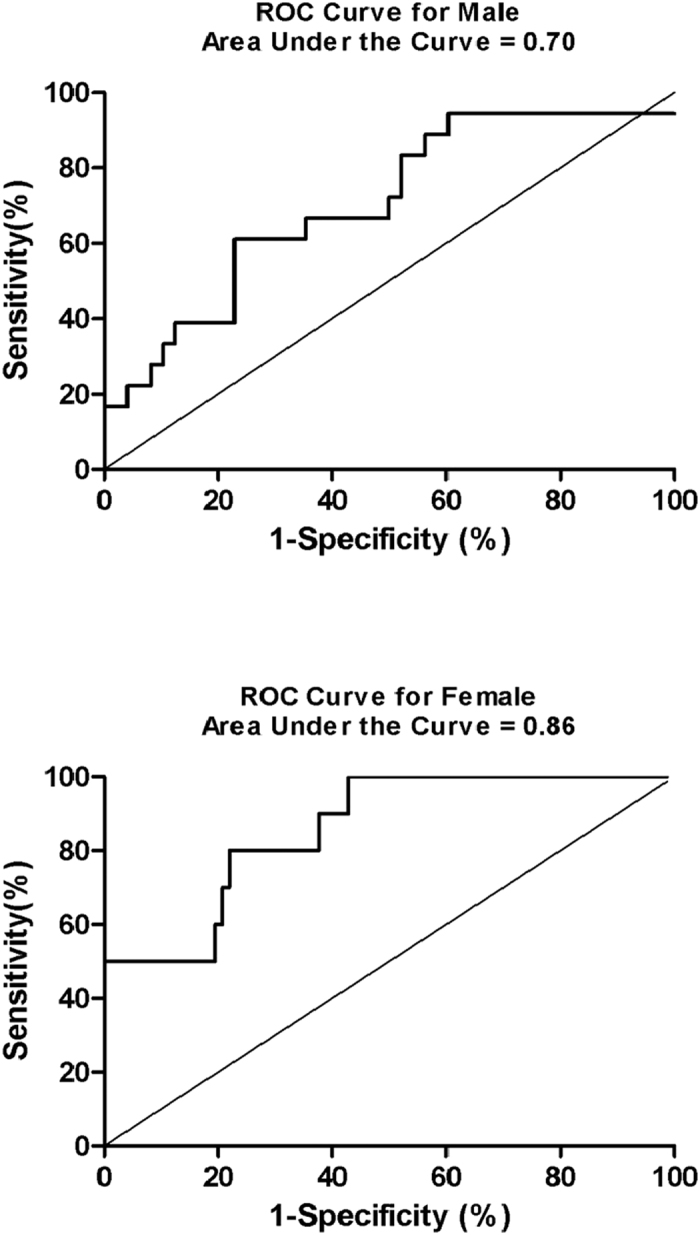
ROC curves for predicting new-onset MetS by ANGPTL8/betatrophin.

According to ROC analysis, The AUC of serum ANGPTL8/betatrophin concentrations were 0.70 (95% CI,0.56–0.85) in males and 0.86 (95% CI, 0.75–0.97) in females (Fig. 2). Based on Youden’s index, our research defined the best cut-off values for serum ANGPTL8/betatrophin concentrations in prediction MetS as 23.9 ng/mL and 31.1 ng/mL for males and females, respectively. The sensitivities and specificities were 61.1%, 77.1% for males, and 80.0%, 77.9% for females, respectively.

Furthermore, we made comparison of ROC curves between ANGPTL8/betatrophin and other metabolic risk parameters (Table 5). It suggested that ANGPTL8/betatrophin is superior to TG, blood pressure, and blood glucose in clinical diagnosis of MetS in females. However, the AUC of ANGPTL8/betatrophin and WC had no significant difference both for males and females.

## Discussion

The data from this prospective cohort study demonstrated that decreased levels of serum ANGPTL8/betatrophin were closely related to the development of MetS in both males and females. The number of MetS components also showed an increasing trend as the serum ANGPTL8/betatrophin levels reduced, suggesting that circulating ANGPTL8/betatrophin may be of importance in the course of MetS and its components.

In the present study, the gender difference in baseline ANGPTL8/betatrophin was significant. Median ANGPTL8/betatrophin level in males was reduced than females (27.05 and 36.68 ng/mL, respectively; *p *< 0.001). Coincidentally, the previous general conclusion was a significant difference in ANGPTL8/betatrophin levels between males and females^9^,^10^,^11^, despite several contrary conclusions^12^. Therefore, our data was stratified by gender. In our study, circulating ANGPTL8/betatrophin levels improved the prediction of newly diagnose high TG in females but not in males. These differences cannot be explained clearly due to the lack of relevant studies.

It is necessary to point that the only MetS component significantly associated with decreased baseline ANGPTL8/betatrophin concentrations is high blood glucose in both males and females. Initially, Melton’s group^6^ reported that the expression of ANGPTL8/betatrophin mRNA was induced by rat models with insulin resistance, and that the overexpression of ANGPTL8/betatrophin itself ameliorated glucose metabolism. In humans, a 70% reduction of ANGPTL8/betatrophin concentrations in T2DM subjects was observed in a previous study^9^. However, Fenzl *et al*. reported that subjects with or without T2DM were not significantly different in the ANGPTL8/betatrophin levels^13^, which is consistent with a recent study^14^. Furthermore, there is accumulating evidences indicating that circulating ANGPTL8/betatrophin concentrations are positively associated with insulin resistance^15^ and diabetes mellitus^16^,^17^,^18^,^19^,^20^. Recently, a meta-analysis reported that subjects with T2DM have higher serum ANGPTL8/betatrophin levels than those without T2DM in non-obese population, but not in the obese population^21^. Hence, it is believed that ANGPTL8/betatrophin may have a dual role in regulating glucose metabolism.

Interestingly, our study pointed out that circulating ANGPTL8/betatrophin levels was correlated inversely with SF and VF, but positive associated with SF/VF in males, indicating that serum ANGPTL8/betatrophin might involve in alteration of fat distribution. Previously, it was reported that ANGPTL8/betatrophin concentrations were negative associated with BMI and fat percentage^11^. On the contrary, Ebert *et al*. observed an elevated expression of ANGPTL8/betatrophin during adipogenesis in 3T3-L1 adipocytes^22^. Thus, there is a complex relationship between ANGPTL8/betatrophin and adipose tissue.

The present study has shown that, in females, a negative relationship between ANGPTL8/betatrophin and the risk of developing high TG remained even after adjustment for visceral fat. A previous study suggested serum levels of TG were significantly reduced in ANGPTL8/betatrophin-null mice compared with wild-type littermates^23^. In 2012, Zhang *et al*. found that overexpression of ANGPTL8/betatrophin in the mouse liver resulted in an elevated TG levels by reduced TG clearance^24^. Furthermore, Fu’s group suggested that ANGPTL8/betatrophin increased lipoprotein lipase inhibition in cardiac and skeletal muscle^25^. In addition, Quagliarini’s group observed that ANGPTL8/betatrophin increased serum TG in a ANGPTL3-dependent manner, and co-expression of ANGPTL8/betatrophin with ANGPTL3 further increased serum TG^26^. In humans, many researches have been performed and the results are controversial. Consistent with our study, Espes’s group has shown a significant negative correlation between ANGPTL8/betatrophin and TG in patients with T2DM^18^. Similar results were also observed in obese individuals and even in lean individuals with normal glucose tolerance^9^,^22^. However, Guo *et al*. indicated that no significant correlation was found between serum ANGPTL/betatrophin levels and lipid profile in 60 normal glucose tolerance individuals and 56 type 2 diabetic individuals^15^, and some other studies suggested that ANGPTL8/betatrophin has a positive correlation with TG^27^,^28^,^29^. It is noteworthy that these researches are biased by different study populations, and the effects of the lipid-lowering drugs on ANGPTL8/betatrophin levels are unknown.

These discrepancies above have sparked a discussion. Fu *et al*. ascribed these conflicting data to the use of different ELISA kits with reactivity to the C- or N-terminus of ANGPTL8/betatrophin^19^. The C-terminal fragments of ANGPTL8/betatrophin can be recognized only by the C-terminal kit, while the full-length ANGPTL8/betatrophin can be measured by either N-terminal or C-terminal kit. That is, the C-terminal kit likely determined total ANGPTL8/betatrophin protein^16^,^30^. However, further investigations are required to definite the biological functions of ANGPTL8/betatrophin in full-length form and its C-terminal fragments. In addition, fasting or fed state^7^,^12^,^26^, and different conditions of blood sample preservation, may also attribute to the discrepancy^31^.

At present, there have been few research studies involving the relationship between circulating ANGPTL8/betatrophin levels and MetS. Recently, in a cross-sectional study, Crujeiras *et al*. observed that an increment of serum ANGPTL8/betatrophin in obese subjects suffering from MetS, as well as a positively association with obesity, lipid profile and glucose metabolism^32^. Despite the lack of relevant studies, accumulating evidence have shown that ANGPTL8/betatrophin is involved in glucose homeostasis and lipid metabolism. Therefore, ANGPTL8/betatrophin might represent a promising diagnostic marker and therapeutic target in MetS.

Several strengths and limitations of the current work should be considered. We investigated the issue based upon a prospective study, which enabled us to establish an independent relationship between baseline concentrations of ANGPTL8/betatrophin and the risk of developing metabolic syndrome. This suggests a possible underlying mechanism for the association between ANGPTL8/betatrophin and risk of MetS and T2DM. However, this study was confined to middle-aged and elderly urban residents, and may not be generalized for other populations. Secondly, we assessed the serum ANGPTL8/betatrophin levels at baseline only, so that the dynamic changes in ANGPTL8/betatrophin cannot be observed. In addition, the present analyses were according to measurements of total serum ANGPTL8/betatrophin levels. It is crucial to determine both the full-length and total ANGPTL8/betatrophin levels to elucidate the functional properties of ANGPTL8/betatrophin.

## Methods

### Study participants

The data from a community-based cohort study of 750 subjects, aged 40–65, who took part in baseline health examinations were collected, in the urban Shenyang area of China, between 2010 and 2013. Based on this cross-sectional study, we excluded the participants who met the following criteria: 1. the presence of metabolic syndrome; 2. a history of malignancy, liver disease, thyroid dysfunction during follow-up; 3. a history of lipid-lowering, anti-diabetes, or antihypertensive drugs during follow-up; 4. the lack of available data on waist circumference, oral glucose tolerance test, blood pressure or lipid profile; 5. missing communication or unwillingness to participate in the follow-up. In the end, 153 subjects (66 males and 87 females) were selected for this study, and follow-up duration was 3.5 years.

This study was approved by the Medical Ethics Committee of the First Affiliated Hospital of China Medical University and was conducted in accordance with approved guidelines and regulations. All subjects from the cohort provided their written informed consent for using their data and blood samples.

### Data collection

At baseline and at endpoint, all the participants underwent comprehensive health examinations. In an interview with trained staff, a standardized questionnaire was completed for each participant, which included demographic and lifestyle characteristics, health status and medical history. The participant’s natural WC was determined. The SBP and DBP of participants were asked to be measured twice by a mercury sphygmomanometer in sitting position on their right arms, after a resting for more than 30 minutes. The mean value from the two measurements was recorded. The height and body weight were measured when the participants wore indoor clothing without shoes. The BMI was estimated according to the following formula: BMI = body weight (kg)/square of height (m^2^).

A venous blood sample was drawn to determine their FPG, lipid profile as well as serum insulin concentrations, and then all study participants accepted an oral glucose tolerance test. The HOMA-IR was evaluated with the formula: HOMA-IR = FPG (mmol/L) × insulin (mIU/L)/22.5. The TC and TG levels were tested by the cholesterol oxidase method and the glycerophosphate oxidase method, respectively. The HDL-c was determined by using an Olympus AU1000 Auto-analyzer (Olympus, Japan). The LDL-c was estimated based on the Friedewald formula. Plasma glucose was determined via the hexokinase method (Roche), and insulin levels were detected by radioimmunoassay (Linco, Inc. St Charles, MO, USA).

Magnetic resonance imaging scans were acquired in the abdominal region, between the 4th and 5th lumbar vertebrae, while the subjects were in the prone position (FOV42 cm * 42 cm, thickness 1.0 cm, six layers; General Electric, Schenectady, NY, USA). SF and VF areas were calculated by two separate technicians using the SLICE-O-MATIC software, version 4.2.

After centrifugation, the supernatant of blood samples was preserved under −80 °C until ANGPTL8/betatrophin analysis in 2014. The concentrations of ANGPTL8/betatrophin were determined using a validated ELSA kit (Human ANGPTL8/betatrophin ELISA kit, Cusabio, Wuhan, China) with intra-assay and inter-assay coefficients of being less than 8% and less than 10%, respectively.

### Metabolic syndrome definition

We identified the newly-diagnosed MetS at follow-up visit, based on the International Diabetes Federation criteria^33^, which included at least three components of the followings: 1. abdominal obesity, defined as a Chinese-specific standard in adults, namely a WC ≥ 90 cm for males or ≥ 85 cm for females^34^; 2. hypertriglyceridemia, defined as a serum level of TG ≥ 1.70 mmol/L; 3. low HDL-c, defined as a serum level of HDL-c < 1.0 mmol/L for men or 1.3 mmol/L for women; 4. hypertension, defined as a SBP ≥ 130 mmHg and/or a DBP ≥ 85 mmHg, or accepting antihypertensive treatments; 5. hyperglycemia, defined as a FPG ≥ 5.6 mmol/L, or a diagnosis of T2DM.

### Statistical analysis

We divided the study cohort into the MetS group and the non-MetS group, based on whether the MetS was developing or not during the follow-up period. For females who had higher levels of ANGPTL8/betatrophin in comparison with males, we performed all analyses separately by gender. Statistical analyses were performed by the SPSS for Windows (version 22.0, SPSS Inc., USA). ROC was analyzed by the MedCalc for Windows (version 16.2, MedCalc Software, Belgium). The data were presented as means ± SD, medians (interquartile range) or counts (percentages) depending upon the different types of the variables. Based on the characteristic of the data, either the *t*-test, the Mann-Whitney U-test or the Chi-squared test were taken in the univariate analysis to estimate the relative elements of the MetS and its components. Logistic regression was performed to analyze the independent correlation between baseline serum concentrations of ANGPTL8/betatrophin and the incidence of newly-onset MetS, and each component during the follow-up period. We adjusted for multiple variables, including baseline age, baseline BMI, baseline LDL-c, baseline TC, baseline HOMA-IR, SF, and VF. In the initial simple regression analysis, the candidate variable was deemed as *p* value less than 0.1, and *p *< 0.05 were defined to be a statistical significance.

## Additional Information

**How to cite this article**: Wang, H. *et al*. The Effects of Serum ANGPTL8/betatrophin on the Risk of Developing the Metabolic Syndrome – A Prospective Study. *Sci. Rep.* **6**, 28431; doi: 10.1038/srep28431 (2016).

## Figures and Tables

**Table 1:** Baseline parameters of study participants according to the MetS status at follow-up.

Characteristic	Male (n = 66)			Female (n = 87)		
	non-MetS	MetS	*p*	non-MetS	MetS	*p*
N (%)	48 (72.7)	18 (27.3)		77 (88.5)	10 (11.5)	
Age (years)	51.1 ± 7.9	50.8 ± 7.2	0.899	51.6 ± 6.0	49.3 ± 5.7	0.261
Current/former smoke n(%)	21 (43.8)	4 (22.2)	0.187	22 (28.6)	4 (40.0)	0.707
Alcohol drinking n(%)	28 (58.3)	10 (55.6)	1.000	34 (44.2)	7 (70.0)	0.229
BMI (kg/m^2^)	23.4 ± 2.6	26.3 ± 2.9	0.000	23.1 ± 2.5	25.0 ± 2.6	0.036
WC (cm)	83.6 ± 7.5	91.7 ± 8.9	0.000	78.4 ± 6.5	83.7 ± 9.4	0.024
WHR	0.88 ± 0.05	0.92 ± 0.06	0.004	0.84 ± 0.05	0.86 ± 0.05	0.266
SBP (mmHg)	121.9 ± 13.5	124.3 ± 11.0	0.499	116.1 ± 12.5	121.1 ± 13.1	0.239
DBP (mmHg)	78.9 ± 8.6	81.5 ± 8.7	0.285	74.5 ± 8.4	80.0 ± 7.9	0.053
FPG (mmol/L)	5.20 ± 0.46	5.39 ± 0.39	0.120	5.26 ± 0.44	5.27 ± 0.32	0.937
2hPG (mmol/L)	5.75 ± 1.61	6.78 ± 1.45	0.020	6.93 ± 1.54	6.28 ± 0.95	0.197
FINS (mIU/L)	16.79 ± 11.11	11.13 ± 5.54	0.051	17.37 ± 6.38	15.83 ± 6.66	0.493
HbA1c (%)	5.55 ± 0.33	5.51 ± 0.51	0.687	5.81 ± 0.44	5.76 ± 0.44	0.747
Triglycerides (mmol/L)	1.1 (0.8–1.5)	1.4 (1.2–2.0)	0.025	1.1 (0.8–1.5)	1.4 (1.0–1.6)	0.353
Cholesterol (mmol/L)	4.73 ± 0.89	5.22 ± 1.01	0.058	5.11 ± 1.01	5.04 ± 0.87	0.846
HDL-c (mmol/L)	1.37 ± 0.30	1.28 ± 0.30	0.306	1.63 ± 0.41	1.38 ± 0.25	0.066
LDL-c (mmol/L)	2.92 ± 0.80	3.45 ± 0.73	0.017	3.03 ± 0.81	3.27 ± 0.74	0.366
ALT (U/L)	23 ± 12	32 ± 31	0.095	17 ± 8	20 ± 16	0.546
AST(U/L)	22 ± 8	26 ± 13	0.080	20 ± 6	21 ± 8	0.669
GGT (U/L)	31 ± 18	36 ± 14	0.321	21 ± 14	25 ± 17	0.422
Log(HOMA-IR) (units)	0.52 ± 0.29	0.36 ± 0.23	0.022	0.58 ± 0.16	0.53 ± 0.22	0.419
Subcutaneous fat (cm^2^)	108.83 ± 45.49	144.44 ± 43.21	0.006	171.63 ± 55.64	197.01 ± 54.05	0.177
Visceral fat (cm^2^)	68.92 ± 40.29	113.89 ± 61.75	0.001	54.35 ± 23.46	70.35 ± 25.43	0.048

BMI, body mass index; WC, waist circumference; WHR, waist-hip ratio; SBP, systolic blood pressure; DBP, diastolic blood pressure; FPG, fasting plasma glucose; 2hPG, two-hour postprandial plasma glucose; FINS, fasting insulin; HDL-c, high-density lipoprotein cholesterol; LDL, low-density lipoprotein cholesterol; ALT, alanine aminotransferase; AST, aspartate transaminase; GGT, glutamyl transpeptidase.

**Table 2:** Baseline serum ANGPTL8/betatrophin levels according to the presence or absence of MetS components during 3.5-year follow-up.

ANGPTL8/Betatrophin (ng/mL)	Male			Female		
	Present	Absent	*p*	Present	Absent	*p*
MetS	22.93 (14.46–30.23)	29.28 (23.92–37.40)	0.011	25.46 (17.96–32.17)	38.10 (31.52–47.79)	0.000
High WC	29.52 (15.53–32.23)	27.02 (21.56–36.24)	0.522	31.57 (25.75–39.52)	37.47 (30.62–48.01)	0.052
Low HDL-c	28.61 (22.17–38.34)	27.05 (20.96–35.88)	0.769	30.43 (27.55–39.52)	37.47 (31.26–47.16)	0.076
High TG	24.14 (15.57–31.74)	27.89 (22.57–36.27)	0.143	35.58 (26.77–44.16)	37.88 (31.09–46.59)	0.258
High BP	25.76 (20.80–31.65)	27.25 (23.90–36.36)	0.344	37.18 (31.52–48.86)	36.60 (26.17–44.42)	0.228
High BG	18.54 (14.47–22.93)	31.01 (26.53–43.98)	0.000	32.59 (23.41–37.32)	39.13 (31.33–48.55)	0.011

TG, triglycerides; BP, blood pressure; BG, blood glucose.

**Table 3:** Odds ratios for new-onset MetS and its components according to baseline serum ANGPTL8/betatrophin levels.

Components of MetS	Male			Female		
	≤ 21.28 ng/mL	>21.28 ng/mL	*p* value	≤ 29.25 ng/mL	>29.25 ng/mL	*p* value
MetS	1.00	0.92 (0.85–0.99)	0.031	1.00	0.84 (0.74–0.96)	0.010
High WC	1.00	0.93 (0.82–1.07)	0.305	1.00	0.98 (0.76–1.24)	0.708
Low HDL-c	1.00	0.91 (0.72–1.15)	0.426	1.00	0.96 (0.78–1.18)	0.706
High TG	1.00	0.86 (0.71–1.04)	0.113	1.00	0.91 (0.84–0.98)	0.013
High BP	1.00	1.00 (0.94–1.06)	0.940	1.00	0.99 (0.94–1.04)	0.707
High BG	1.00	0.74 (0.57–0.96)	0.021	1.00	0.85 (0.76–1.01)	0.014

Adjusted for age, baseline BMI, LDL-c, TC, HOMA-IR, SF and VF.

**Table 4:** The area under ROC curves of ANGPTL8/betatrophin and other metabolic parameters in prediction of MetS.

	Male		Female	
	AUC	*p*	AUC	*p*
ANGPTL8/betatrophin	0.70 ± 0.07	0.011	0.86 ± 0.06	<0.001
Waist	0.77 ± 0.07	<0.001	0.71 ± 0.10	0.033
HDL-c	0.59 ± 0.08	0.255	0.69 ± 0.08	0.054
TG	0.68 ± 0.07	0.025	0.59 ± 0.09	0.355
SBP	0.58 ± 0.07	0.328	0.63 ± 0.09	0.175
DBP	0.56 ± 0.08	0.494	0.65 ± 0.07	0.116
FPG	0.63 ± 0.08	0.096	0.54 ± 0.09	0.695
2hPG	0.67 ± 0.07	0.033	0.37 ± 0.08	0.194

**Table 5:** Comparison of the area under curves between ANGPTL8/betatrophin and other metabolic parameters in prediction of MetS.

	Male			Female		
	AUC_ANGPTL8_-AUC	Z statistic	*p*	AUC_ANGPTL8_-AUC	Z statistic	*p*
Waist	−0.07 ± 0.09	0.749	0.454	0.15 ± 0.11	1.405	0.160
HDL-c	0.11 ± 0.09	1.209	0.227	0.17 ± 0.11	1.598	0.110
TG	0.02 ± 0.11	0.213	0.831	0.27 ± 0.11	2.782	0.005
SBP	0.13 ± 0.09	1.343	0.179	0.23 ± 0.09	2.405	0.016
DBP	0.15 ± 0.11	1.342	0.179	0.20 ± 0.09	2.269	0.023
FPG	0.07 ± 0.11	0.645	0.519	0.32 ± 0.11	2.773	0.006
2hPG	0.03 ± 0.11	0.295	0.768	0.23 ± 0.11	2.134	0.033
